# Abiraterone shows alternate activity in models of endocrine resistant and sensitive disease

**DOI:** 10.1038/s41416-018-0158-y

**Published:** 2018-07-11

**Authors:** Nikiana Simigdala, Sunil Pancholi, Ricardo Ribas, Elizabeth Folkerd, Gianmaria Liccardi, Joanna Nikitorowicz-Buniak, Stephen R. Johnston, Mitch Dowsett, Lesley-Ann Martin

**Affiliations:** 1 The Breast Cancer Now Toby Robins Research Centre, The Institute of Cancer Research, London, SW7 3RP UK; 2 The Ralph Lauren Centre for Breast Cancer Research, The Royal Marsden Hospital, London, SW3 6JJ UK; 3 Breast Unit, The Royal Marsden Hospital, London, SW3 6JJ UK

## Abstract

**Background:**

Resistance to endocrine therapy remains a major clinical problem in the treatment of oestrogen-receptor positive (ER+) breast cancer. Studies show androgen-receptor (AR) remains present in 80–90% of metastatic breast cancers providing support for blockade of AR-signalling. However, clinical studies with abiraterone, which blocks cytochrome P450 17A1 (CYP17A1) showed limited benefit.

**Methods:**

In order to address this, we assessed the impact of abiraterone on cell-viability, cell-death, ER-mediated transactivation and recruitment to target promoters. together with ligand-binding assays in a panel of ER+ breast cancer cell lines that were either oestrogen-dependent, modelling endocrine-sensitive disease, or oestrogen-independent modelling relapse on an aromatase inhibitor. The latter, harboured wild-type (wt) or naturally occurring *ESR1* mutations.

**Results:**

Similar to oestrogen, abiraterone showed paradoxical impact on proliferation by stimulating cell growth or death, depending on whether the cells are hormone-dependent or have undergone prolonged oestrogen-deprivation, respectively. Abiraterone increased ER-turnover, induced ER-mediated transactivation and ER-degradation via the proteasome.

**Conclusions:**

Our study confirms the oestrogenic activity of abiraterone and highlights its differential impact on cells dependent on oestrogen for their proliferation vs. those that are ligand-independent and harbour *wt* or mutant *ESR1*. These properties could impact the clinical efficacy of abiraterone in breast cancer.

## Introduction

Endocrine therapies have been very successful in treating oestrogen receptor-positive (ER+) breast cancer, which accounts for over 70% of all breast cancers. However, almost 40% of patients relapse on therapy.^1–6^ Identification of potential resistance mechanisms associated with either de novo or acquired resistance is therefore of paramount clinical importance. Unfortunately, cancer is a very heterogeneous disease^7–11^ and depending on the patient and the cancer genetic/epigenetic landscape, multiple resistance mechanisms can occur (reviewed by ref.^4^), suggesting that one drug cannot fit all.

The androgen receptor (AR) is expressed in 50–70% of all breast cancer. In particular 80–90% of ER+ breast cancer also express AR which associates with a more favourable prognosis.^12,13^ Nonetheless, pre-clinical studies indicate increased abundance of AR reduces response to both tamoxifen and oestrogen-deprivation^14,15^ an observation that has been supported in retrospective clinical investigations.^14,16^ Furthermore, pre-clinical studies have suggested the potential usefulness of therapies that target AR signalling in breast cancer^16^ and have paved the way for a number of clinical trials.^17–19^

Several drugs are used to block AR action: enzalutamide and bicalutamide that function as AR-antagonists, enobosarm that acts as a selective AR modulator (SARM) and abiraterone, a steroidal compound that blocks CYP17A1.^19–21^

However, clinical studies in breast cancer to date have shown mixed response to blockade of AR signalling. For instance, enobosarm provided clinical benefit in 6/17 ER+ metastatic breast cancer patients,^22^ while abiraterone showed no improvement in progression-free survival (PFS) in a similar cohort.^18^

A recent in vitro study showed abiraterone acted as an agonist in ER+ breast cancer cells and elicited ER-transcriptional activity.^23^ Such oestrogenic activity could impact on the effectiveness of abiraterone in the clinic given the proliferative response to oestrogen signalling by most ER+ breast cancer. However, the cell line model used by Capper et al.^23^ was representative of the endocrine therapy naïve setting and therefore did not recapitulate the clinical scenario in which the drug is currently being tested. In order to address the potential mechanism underlying the lack of clinical benefit gained from abiraterone in ER+ metastatic breast cancer patients, we investigated its impact on ER signalling in a panel of ER+ breast cancer cell lines sensitive (i.e., dependent on oestrogen for their proliferation) or resistant to long-term oestrogen-deprivation (LTED), modelling relapse on an aromatase inhibitor (AI), and harbouring various naturally occurring *ESR1* mutations. Our data indicate that abiraterone may have context-dependent roles in ER+ breast cancer that can be influenced by prior hormonal therapies and that *ESR1* mutation status may influence its efficacy in the clinical setting.

## Materials and methods

### Cell culture

All wild type (wt) cell lines (MCF7, HCC1428 and SUM44) were purchased from ATCC or Asterand. Upon receipt cells were aliquoted to prevent phenotypic drift and authenticated by STR. All cell lines were routinely screened for mycoplasma infection. The wt cell lines were cultured in phenol red-free RPMI 1640 (Gibco, Thermo Fisher Scientific, UK) supplemented with 10% FBS (Gibco, Thermo Fisher Scientific, UK) and 1 nM 17-β-estradiol (E2) (Sigma-Aldrich, UK). Long-term oestrogen deprived (LTED) derivatives of the cell lines were cultured in phenol red-free RPMI 1640 in the absence of exogenous E2 and supplemented with 10% dextran charcoal-stripped bovine serum (referred to as 10% DCC medium).^24^ Two MCF7-LTED derivatives were used within the study one harbouring wt-ESR1 (MCF7-LTED^wt^), the other harbouring a naturally occurring *ESR1*^*Y537C*^ mutation (MCF7-LTED^Y537C^). Additionally SUM44-LTED express an *ESR1*^*Y537S*^ mutation (SUM44-LTED^Y537S^) whilst HCC1428-LTED contain wt-ESR1.^25^

### Proliferation assays

Wt cell lines were stripped of E2 by culturing in 10% DCC medium for 5 days, with daily medium changes. LTED and stripped wt cell lines were seeded into 96-well tissue culture plates (Greiner one, UK) in 10% DCC medium, as previously described.^26^ Cells were allowed to acclimatise overnight. Monolayers were treated with escalating concentrations of abiraterone (CAS no-S1123, SelleckChem, UK), E2, ICI (ICI 182,780) (CAS no 129453-61-8, SelleckChem, UK) or combinations of the agents over a 6-day period. Treatment medium was replenished on day 3. Cell viability was determined using CellTitre-Glo® Luminescent Cell Viability Assay (Promega), according to the manufacture’s protocol. Luminescent signal was read on Victor spectrophotometer (Perkin Elmer, Wokingham, UK). Values were expressed as percent (%) viable cells relative to the vehicle treated control. Statistical analysis was performed in Prism using non-linear fit curve, two-way ANOVA and multiple comparison analysis using Dunnett test. For the siRNA knockdown assays, cells were seeded in 10% DCC and transfected with sicontrol (non-targeting pool) or siRNA targeting *ESR1* or *AR* (ON-TARGETplus siRNA, GE Dharmacon) using lipofectamine RNAimax (Invitrogen, Grand Island, NY, USA),^26^ according to the manufacturer’s protocol. After 24 h, monolayers were then treated with 10% DCC plus or minus oestrogen (1 nM) or abiraterone (7.5 μM) and cells cultured for a total of 6 days. The efficiency of the knock down was assessed by quantitative reverse transcription polymerase chain reaction (qRTPCR). Cell viability was determined using the CellTitre-Glo® Luminescent Cell Viability Assay (Promega). Values were expressed as fold-change relative to the vehicle treated control. Statistical analysis was performed using Student’s *t*-test.

### Live cell imaging

In order to monitor proliferation over time live cell imaging was conducted using Incucyte S3 Live-Cell Analysis System (Essen Bioscience). In brief, cells were stripped of E2 as described above and seeded into 96-well tissue culture plates. Drugs were administered and phase-contrast images of the wells were acquired every 4 h in real time over 7 days. Masks were created using Incucyte S3 software to quantitate percentage phase-contrast confluence for each well and quantified time-lapse curves were generated. Data represent mean of 6–8 replicates (16 images per time point). Statistical analysis was performed in Prism using 2-way ANOVA.

### Proliferation assay in spheroids

HCC1428-LTED spheroids, were generated by seeding 2000 cells in 10% DCC medium, into ultralow attachment 96-well plates (Corning, UK) and centrifuged for 10 min at 900 rpm. After 3 days, spheres were treated with escalating doses of abiraterone and re-treated every 3–4 days. After 10 days of treatment images of spheres were taken using CeligoS (Nexcelome Bioscience, Lawrence, MA, USA) and viability was assessed using CellTitre-Glo® Luminescent 3D Cell Viability Assay (Promega, UK).

### Transcription assays

Stripped wt cells and LTED cells were seeded into 24-well plates^27^ in 10% DCC medium. The following day, the cells were transfected with an oestrogen response element linked luciferase (EREtkLuc) and β-galactosidase reporter-constructs using Fugene® 6 (Promega).^27^ The following day, cells were treated for the respective treatments highlighted within the figure legends for 24 h. The luciferase activity (Promega) and β-galactosidase (GalactoStar; Applied Biosystems) were measured using a luminometer. Each experiment was performed in triplicate and the luciferase values were normalised to the β-galactosidase. For the proteasome inhibition experiments, the cells were treated for 16 h as previously described.^28^ Statistical analysis was performed using Student’s *t*-test.

### Ligand binding assays

Stripped wt-MCF7 and MCF7-LTED^Y537C^ cells were seeded into 12 well plates at a density of 2.5 × 10^4^ cells per well in 10% DCC medium and allowed to acclimatise for 24 h. The following day, cells were transferred to serum free medium for a further 24 h. Subsequently, cells were treated in competition assays with 2 nM ^3^H-E2^29^ and escalating concentrations of E2 or abiraterone. Monolayers were incubated for 2 h at 37 °C, washed twice with cold phosphate buffered saline (PBS) and lysed in 250 μl of protein lysis buffer. Radioactivity was measured (1900CA analyser, Perkin Elmer).^30^ Experiments were conducted in triplicate. Prism was used to calculate Ki and IC_50_ using non-linear regression analysis.

### Western blot analysis

Protein extracts were generated, as previously described.^31^ Equal amounts of protein were resolved by SDS-PAGE and subjected to immunoblot analysis. Antigen–antibody interactions were detected with ECL-reagent (Amersham, Amersham, UK). Proteins were detected using the following antibodies: total-ESR1 (Santa-Cruz sc8002), PGR (Novocastra NCL-L-PGR), TFF1 (ab92377), GREB1 (ab72999), AR (CST-5153), CCND3 (CST-2936), CDK4 (CST-2906), pRBser780 (CST-3590), pRBser807 (CST-9308), p130 (sc-317), p107 (sc-318), total-RB (CST-9309), and tubulin (Sigma T-9026). Secondary antibodies were used at concentration of 1/2000 (Dako, Denmark A/S). MG-132 was purchased from Selleckchem (S2619) and was used at 10 μM final concentration.

### Tandem ubiquitin-binding entity (TUBE)

Cells were lysed on ice in lysis buffer [200 mM Tris HCL pH 7.5, 150 mM NaCl, 2 mM EDTA, 10% Glycerol, 50 mM NaF, 200 μM NaVO_4_, complete protease inhibitor cocktail EDTA-free from Roche, supplemented with fresh PR619 (DUB inhibitor, 1:2000, SI9619 LifeSensors). Supernatant was collected after 10 min centrifugation in a cooling centrifuge. Protein lysate was incubated together with pre-washed glutathione sepharose beads (GST Sepharose 4B, GE Healthcare) and recombinant ubiquitin in a rocker overnight at 4 °C. The next day, the samples were washed five times with washing buffer (PBS, 1% Triton, 1 mM EDTA plus PR619) and centrifuged each time at 4000 rpm for 30 s at 4 °C. After complete removal of the last wash by aspiration, samples were boiled using laemmli (plus DTT) and subjected to SDS-PAGE and Western blot analysis. Primary antibodies used were; ER (Santa-Cruz sc8002) and ubiquitin (P4D1, CST-3936). Secondary antibodies were used at concentration of 1/2000 (Dako, Denmark A/S).

### qRT-PCR

RNA was extracted 24 h after treatments using RNeasy columns and quantification was performed using a NanoDrop 1000 spectrometer. cDNA was generated using SuperScript III First Strand Synthesis System for RT-PCR (Invitrogen, Grand Island, NY, USA). cDNA was subjected to quantitative PCR (Applied Biosystems). Taqman gene expression assays (Applied Biosystems) was used *ESR1* (Hs00907239_m1), *AR* (Hs00171172_m1) and *FKBP15* (Hs00391480_m1) as housekeeping gene, to normalise the data. The relative quantity was determined using ΔΔ*C*t.

### ChIP qPCR

ChIP experiments were performed, as previously described.^32^ Briefly, cells were synchronised with α-amanitin,^33^ then treated for 45 min with E2 (1 nM), ICI (10 nM), abiraterone (7.5 μM) or combination and fixed. The antibodies used were anti-ER (Santa Cruz Biotechnology; sc-543×), anti-CBP (Santa Cruz Biotechnology; sc-369×) and Mouse IgG1 (Dako, Denmark A/S). The resulting DNA was subjected to quantitative PCR analysis using SYBR green (Applied Biosystems) with the following primers for *TFF1*: (forward) 5′-GGC CAT CTC TCA CTA TGA ATC ACT TCT GCA-3′ and (reverse) 5′-GGC AGG CTC TGT TTG CTT AAA GAG CGT TAG-3′, and for *GREB1*: (forward) 5′-GAA GGG CAG AGC TGA TAA CG 3′ and (reverse) 5′-GAC CCA GTT GCC ACA CTT TT.

### Cell death assay

Cell death assays were carried out as previously described.^34^ In brief, cells were seeded in 96 well plates and 24 h later were treated with increasing doses of abiraterone or E2. The treatment was replaced after 3 days and cells were cultured for a total of 6 days. PI was added after 6 days. Cell death was analysed by flow cytometry quantification of PI (2 μg/mL) uptake using a FACSCalibur (BD Biosciences). Statistical analysis was performed using one-way ANOVA with Bonferroni correction.

## Results

### The differential impact of abiraterone on the proliferation of cell lines resistant to LTED

**Fig. 1:**
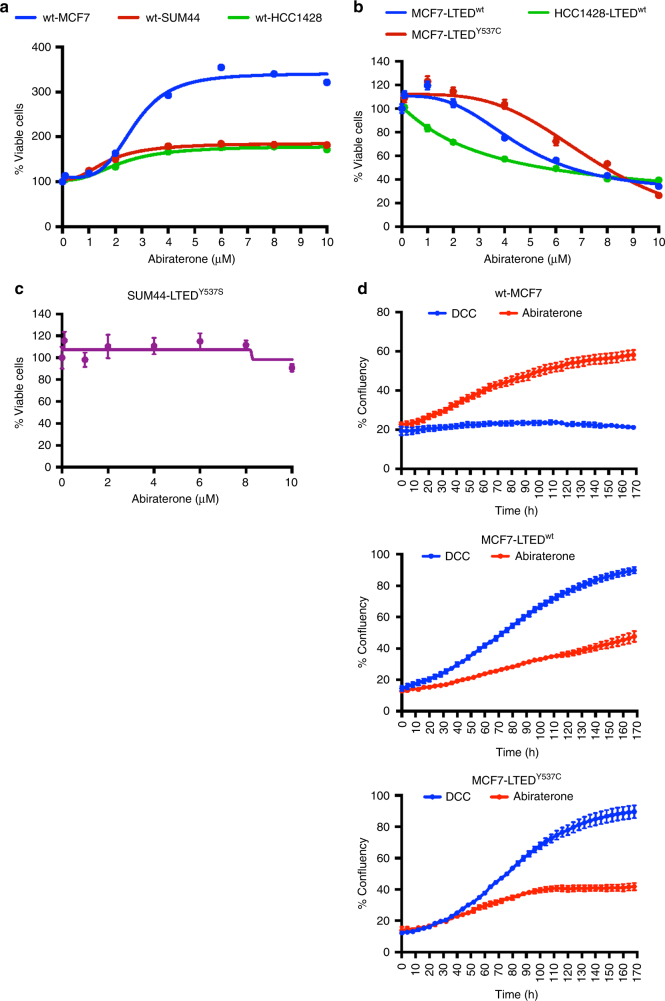
Alternate sensitivity to abiraterone in ER+ breast cancer cell line models. **a** Wt-MCF7, wt-HCC1428, wt-SUM44 and **b** MCF7-LTED^wt^, MCF7-LTED^Y537C^, HCC1428-LTED and **c** SUM44-LTED^Y537S^ cell lines were treated for 6 days with escalating concentrations of abiraterone (0.1–10 μM). The concentration range used was based on previous published studies. Cell viability was measured using TitreGlo and expressed as percent viable cells compared to vehicle treated control. Wt lines were grown in hormone-deprived conditions for 5 days prior to treatment with abiraterone. **d** Time course analysis of proliferation in response to abiraterone (7 μM) assessed using incucyte over a period of 7 days with 4 hourly readings. Data is expressed as percentage of confluency. The data shown is representative of two independent biological experiments with six to eight replicates per treatment. Bars represent ±SEM

Given the recent findings suggesting abiraterone can induce growth in ER+ breast cancer cells in vitro,^23^ we assessed the impact of abiraterone on the proliferation of a panel of cell lines modelling both oestrogen-dependent and independent growth (Fig. 1).^31^ Wt cell lines were grown in the absence of E2 for 5 days prior to the treatment with escalating concentrations of abiraterone. In order to be consistent with the abiraterone concentrations used in previous studies, we adopted a concentration range of abiraterone from 0.1–10 μM.^23,30^

In keeping with previous findings,^23^ all wt ER+ breast cancer cell lines tested showed a concentration-dependent increase in proliferation in response to abiraterone (wt-MCF7 EC_50_ 2.76 μM (95% CI 2.60–2.94 μM); wt-SUM44 EC_50_ 1.56 μM (95% CI 1.4–1.72 μM); wt HCC1428 EC_50_ 2.06 μM (95% CI 1.81–2.32 μM)) (Fig. 1a). Contrastingly, two MCF7-LTED models harbouring either ESR1^wt^ or ESR1^Y537C^ as well as HCC1428-LTED showed over a 50% reduction in proliferation in the same concentration range tested (MCF7-LTED^Y537C^ IC_50_ 5.79 μM (95% CI 5.41–6.18 μM); MCF7-LTED^wt^ IC_50_ 3.83 μM (95% CI 3.54–4.13 μM and HCC1428-LTED IC_50_ 1.98 μM (95% CI 1.75–2.22 μM)) (Fig. 1b). However, SUM44-LTED^Y537S^ cells were resistant to the anti-proliferative effect of abiraterone (Fig. 1c).

The contrasting effect of abiraterone was confirmed in MCF7 isogenic models using real-time imaging of cell growth over a period of 7 days (Fig. 1d). Furthermore, the antiproliferative effect of abiraterone within the LTED cells was confirmed in 3D spheroids (Supplementary Figure S1).

As abiraterone is known to have a steroidal structure,^21^ we hypothesised the drug may directly bind to ER. In order to address this, we conducted competitive ligand binding assays. The Ki for wt-MCF7 in response to E2 was 0.16 nM (95% CI 0–1.58) and for MCF7-LTED^Y537C^ 0.27 nΜ (95% CI 0.09–0.6). Binding of abiraterone demonstrated a Ki of 0.39 μM (95% CI 0.1–1.2) for wt-MCF7 and 0.25 μM (95% CI 0–3.6) for MCF7-LTED^Y537C^ (Supplementary Table S1).

**Fig. 2:**
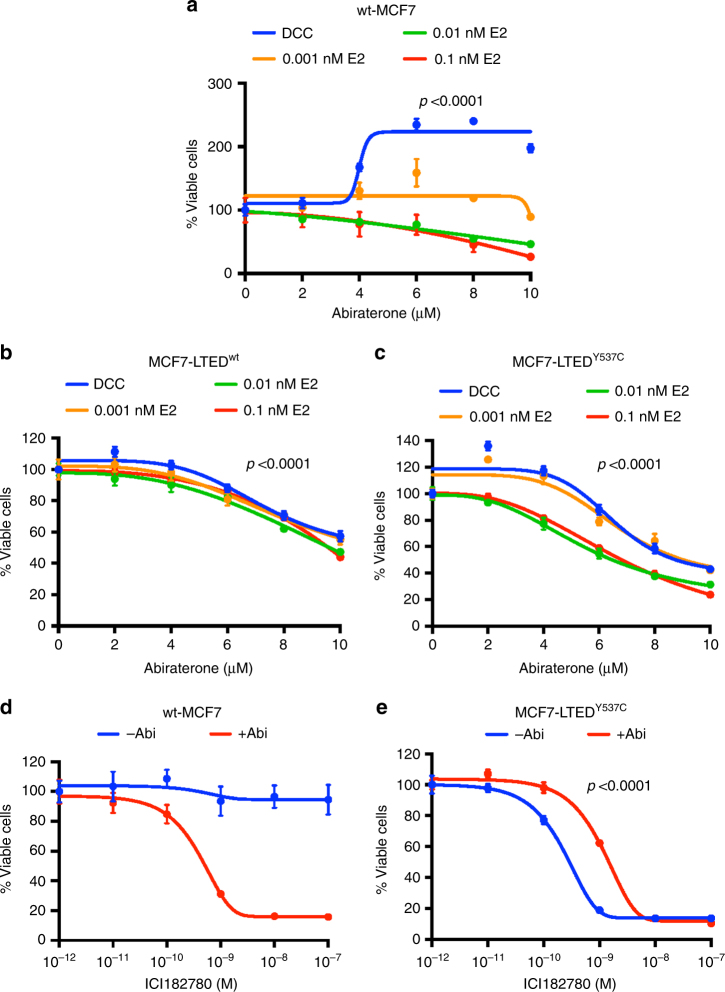
Abiraterone exhibits oestrogenic activity in wt-MCF7 and MCF7-LTED. Wt-MCF7 cells were grown in hormone-deprived conditions prior to treatment. **a** Abiraterone (Abi) alone or in combination with three concentrations of E2 (0.001, 0.01 and 0.1 nΜ). **b** MCF7-LTED^wt^ or **c** MCF7-LTED^Y537C^ were treated with escalating concentrations of abiraterone (Abi) alone or in combination with three concentrations of E2. **d** Wt-MCF7 or **e** MCF7-LTED^Y537C^ were treated with increasing doses of ICI 182,780 (ICI) alone, or with 7.5 μM Abi. Cell viability was measured using TitreGlo and expressed as fold change relative to vehicle treated control. Data shown is representative of two biological experiments and eight replicates per treatment. Bars represent ±SEM. Statistical analysis was performed in Prism using two-way ANOVA and Dunnett’s test

To assess the apparent context-specific agonist and antagonist nature of abiraterone, wt-MCF7 and MCF7-LTED cell line models were treated with three concentrations of E2 (0.001, 0.01 and 0.1 nΜ), escalating concentrations of abiraterone alone or in combination (Fig. 2a–c). The agonist activity of abiraterone in wt-MCF7 was decreased by the addition of E2 in a dose-dependent manner (Fig. 2a) (adj. *p* < 0.0001 Dunnett test). Contrastingly, abiraterone alone caused a concentration-dependent decrease in the cell viability of MCF7-LTED^wt^ and MCF7-LTED^Y537C^, which was enhanced by the addition of E2 (adj. *p* < 0.0001, Dunnett test) (Fig. 2b, c).

To explore the oestrogenic activity of abiraterone, wt-MCF7 and MCF7-LTED^Y537C^ were treated with ICI, a drug that degrades ER, in the presence or absence of abiraterone. As expected, the wt-MCF7, in the absence of ligand, a setting in which ER is not cycling on promoters, showed no response to escalating concentrations of ICI, however, addition of abiraterone resulted in a marked decrease in proliferation (IC_50_ = 0.45 nM) (Fig. 2d). In contrast MCF7-LTED^Y537C^, which are oestrogen-independent showed a dose-dependent decrease in proliferation in response to ICI alone (IC_50_ = 0.23 nM) supporting their ligand-independent phenotype. Addition of abiraterone shifted the dose response curve to the right (IC_50_ = 1.3 nM) suggesting abiraterone antagonised the anti-proliferative effect of ICI (Fig. 2e).

Taken together, these data suggest that the weak oestrogenic activity of abiraterone, potentially given its steroidal structure, may impact on ER-signalling, in a context-specific manner depending on the history of the cells and the *ESR1* mutation status.

### ER-activity is increased in the presence of abiraterone in both hormone-dependent and hormone-independent cell lines

**Fig. 3:**
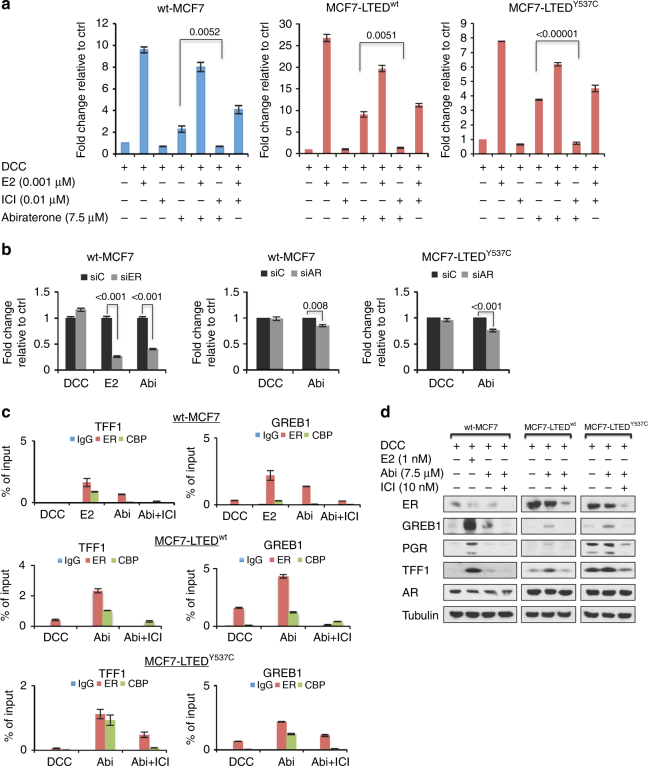
ER-mediated transactivation is increased in the presence of abiraterone and the action is reversed by ICI. **a** wt-MCF7, MCF7-LTED^wt^ or MCF7-LTED^Y537C^ were transfected with an ERE-luciferase (EREII-tk-luc) reporter construct and treated with E2, abiraterone (Abi), ICI 182,780 (ICI) alone or in combination. Data shown is representative of two biological experiments and three replicates per treatment. Bars represent ±SEM. **b** Viability assays on wt-MCF7 and MCF7-LTED^Y537C^ in the presence of siRNA for control, ER and AR with or without abiraterone (Abi). **c** Chromatin immunoprecipitation (ChIP) of ER and its co-factor CBP was carried out in synchronised wt-MCF7, MCF7-LTED^wt^ and MCF7-LTED^Y537C^. IgG was used as a negative control. The cells were synchronised with α-amanitin and treated for 45 min with Abiraterone (Abi), ICI 182,780 (ICI) or in combination. Recruitment of ER and CBP was assessed in the *GREB1* and *TFF1* promoters. Data shown is representative of three technical replicates. Bars represent ±SEM. **d** Protein levels of oestrogen-regulated genes, GREB1, PGR and TFF1 together with ER and AR were assessed in the presence of abiraterone (Abi) (7.5 μM) for 48 h in wt-MCF7, MCF7-LTED^wt^ and MCF7-LTED^Y537C^

To address the impact of abiraterone on ER-mediated transactivation, in the MCF7-LTED models, cells were transfected with an oestrogen-response-element (ERE) linked-luciferase reporter and exposed to escalating concentrations of abiraterone. ER-mediated transactivation was increased in a concentration-dependent manner, while ER-protein levels were decreased (Supplementary Figure S2A & B). We next assessed the relative effect of abiraterone and E2 on ER-transactivation in the MCF7 isogenic models (Fig. 3a). As expected, E2 caused a dramatic rise in ERE-linked luciferase activity, which was suppressed by the addition of ICI. Abiraterone also increased ER-mediated transcription 2-fold in wt-MCF7, 9-fold in the MCF7-LTED^wt^ and 4-fold in MCF7-LTED^Y537C^ (Fig. 3a). Of note, the combination of abiraterone and E2 appeared to antagonise E2-mediated transcription although this did not meet statistical significance (*p* > 0.1). The effect of single agent abiraterone was antagonised by the addition of ICI in all cell lines tested, causing a greater than 4-fold reduction in ER-activity (*p* < 0.005). These data were confirmed in HCC1428-LTED but not SUM44-LTED cells which harbour the *ESR1*^*Y537S*^ mutation (Supplementary Figure S2C).

The impact of abiraterone on ER was also assessed in a viability assay where ER was knocked down in wt-MCF7 in the absence of ligand or in the presence of E2 or abiraterone (Fig. 3b and Supplementary Figure S3). In this setting, siER caused 74% drop in the viability of wt-MCF7 in the presence of E2 (*p* < 0.001) and 60% drop in the presence of abiraterone (*p* < 0.001). As expected in the absence of ligand (DCC medium), a setting in which ER is no longer cycling, siER had no impact on viability. This result was not recapitulated when the AR was knocked down either in the wt-MCF7 or MCF7-LTED^Y537C^ (Fig. 3b and Supplementary Figure S3). This suggests that abiraterone, similar to E2, allows ER to cycle on promoters but does not impact on AR in these models.

To further assess the impact of abiraterone on ER-transactivation, chromatin immunoprecipitation (ChIP) of ER and its co-activator CREB binding protein (CBP) were conducted in synchronised wt-MCF7, MCF7-LTED^wt^ and MCF7-LTED^Y537C^ in response to abiraterone or abiraterone plus ICI. Recruitment of ER and CBP was enriched in response to abiraterone on both *TFF1* and *GREB1* promoters (Fig. 3c) and a concordant increase in protein abundance was observed for TFF1 and GREB1 in all MCF7 cell lines tested. PGR increased significantly in wt-MCF7 cells in response to abiraterone and to a lesser degree in MCF7-LTED^Y537C^. As expected, MCF7-LTED^wt^ cells, which express very low levels of PGR, showed the least response to abiraterone. Noteworthy, no impact on AR was evident (Fig. 3d). In further support, addition of ICI caused a reduction in ER and CBP recruitment (Fig. 3c).

In summary, these data provide further support suggesting abiraterone can bind the ER complex and enhance its activity.

### Abiraterone-induced ERα-transactivation is dependent on classical proteasome-mediated degradation

**Fig. 4:**
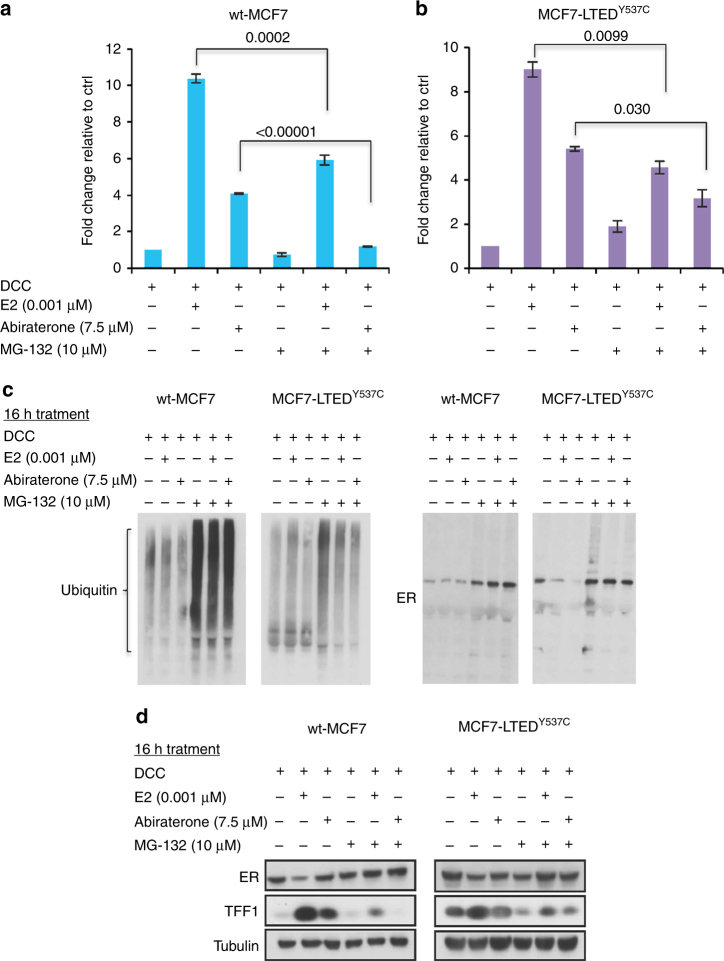
Abiraterone, similarly to E2, facilitates ER-transactivation and ER-degradation via the proteasome. **a** wt-MCF7 (**b**) and MCF7-LTED^Y537C^ were transfected with the EREII-tk-luc construct and treated for 16 h with E2, abiraterone, ICI or in combination in the presence or absence of MG-132 (10 μM). Data is representative of two biological experiments with three replicates for each treatment. Bars represent ±SEM. **c** Tandem ubiquitin-binding entity (TUBE) followed by immunoblot detection of ubiquitin and ER was carried out in wt-MCF7 and MCF7-LTED^Y537C^, treated for 16 h with E2 or abiraterone in the presence or absence of MG-132 (10 μM). **d** Concomitant Western blot analysis of ER and TFF1 assessed after 16 h treatment with E2 or abiraterone in the presence or absence of MG-132 (10 μM)

The transcriptional activity of ER is dependent on proteasome function leading to the cyclical binding of ER on ERE within target genes.^28,35^ Since abiraterone appeared to enhance ER-mediated transcription, we assessed its impact on ER-proteasomal degradation. Addition of the proteasome inhibitor MG-132 caused a 50% reduction in E2 (*p* = 0.0002) and a 70% decrease in abiraterone ER-mediated transactivation (*p* < 0.00001) (Fig. 4a, b). As ER levels drop in response to ligand binding, we conducted tandem ubiquitin binding entity (TUBE),^36^ in order to confirm the association of ER with ubiquitin in response to ligand. Cell lines were treated with E2, abiraterone alone or in combination with MG-132. As expected, capture of ubiquitylated proteins showed enrichment in response to MG-132 (Fig. 4c). Immunoblotting for un-modified ER showed enrichment with abiraterone or E2 in combination with MG-132 (Fig. 4c). Furthermore, addition of MG-132 also reduced the abundance of TFF1 (Fig. 4d). Taken together, these data suggest the ability of abiraterone to promote ER-activity via ligand-dependent proteasome-mediated cycling.

### Abiraterone showed alternate effects on cell cycle in wt-MCF7 and LTED model systems

**Fig. 5:**
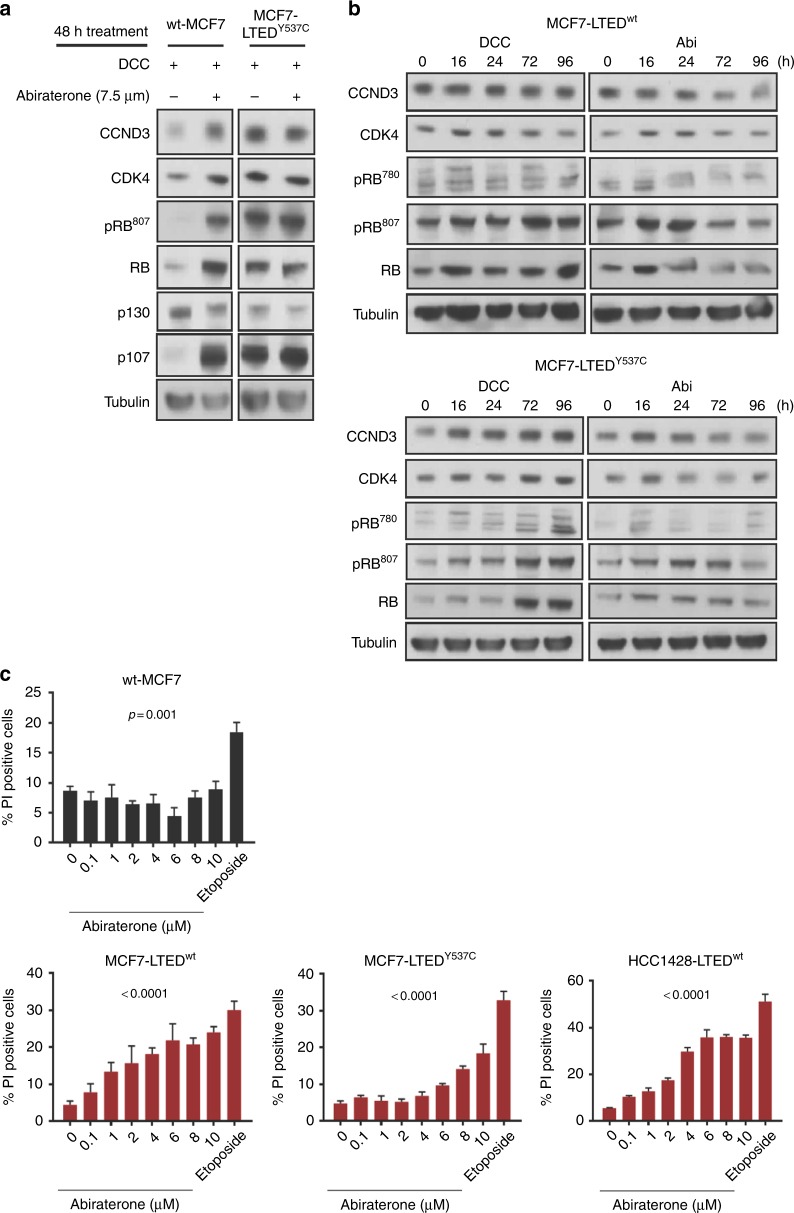
Abiraterone causes a drop in cell cycle markers and induces cell death. **a** The protein levels of cell cycle markers (CCND3, CDK4, pRB^807^, RB, p130 and p107) were assessed in wt-MCF7 and MCF7-LTED^Y537C^ in the presence or absence of abiraterone (7.5 μM) after 48 h treatment. **b** Time course assessment of cell cycle markers (CCND3, CDK4, pRB^780^, pRB^807^, RB) in MCF7-LTED^wt^ and MCF7-LTED^Y537C^ in the presence of abiraterone (Abi) (7.5 μM) for 96 h. **c** Quantification of PI-positive cells (wt-MCF7, MCF7-LTED^wt^, MCF7-LTED^Y537C^, HCC1428-LTED) treated with escalating doses of abiraterone for 6 days. Etoposide was used as a positive control (50 μM)

As abiraterone appeared to have differential effects on cell proliferation in the wt-MCF7 and MCF7-LTED models, we assessed its impact on cell cycle markers of the G1/S phase after 48 h treatment with abiraterone. In wt-MCF7 cells, previously stripped of E2, abiraterone treatment increased abundance of CCND3, CDK4, pRB and p107 indicative of cell cycle progression. Contrastingly, no impact was evident on these cell cycle regulatory proteins in the MCF7-LTED cells after 48 h. To address this further we carried out a time course over 96 h. Abiraterone appeared to have little impact on cell cycle over the first 24–48 h. However, at 72 h posttreatment a reduction in CCND3, total RB and pRB was evident (Fig. 5a, b).

Previously, we and others^37–41^ have shown that high concentrations of E2, which elicit maximum proliferation of the wt-ER+ cell lines, produce an anti-proliferative effect in some ER+ LTED models, an observation that we recapitulated in this study (Supplementary Figure S4). Contrastingly SUM44-LTED, which harbour an *ESR1*^*Y537S*^ mutation, showed no response to either abiraterone (Fig. 1c) or E2 (Supplementary Figure S4).

We hypothesised that abiraterone may have a similar impact. We therefore treated wt-MCF7 and both MCF7-LTED models with escalating concentrations of abiraterone for 6 days and determined cell death using propidium iodide exclusion. As expected, abiraterone caused a concentration-dependent increase in cell death in the MCF7-LTED. This observation was confirmed in a second model, HCC1428-LTED (Fig. 5c).

These findings suggest that abiraterone exerts stimulatory or inhibitory effects in ER+ breast cancer, depending on whether the cells are oestrogen-dependent (AI-sensitive) or oestrogen-independent (AI-resistant) but also that context in which resistance occurs can influences response to abiraterone.

## Discussion

Despite the great efficacy of endocrine agents in treating ER+ breast cancer patients, relapse and outgrowth of cancer cells occurs in many patients with primary disease and invariably in metastatic disease.^42^ Therefore, identification of new treatment options is of paramount importance. Previous studies in prostate cancer have shown that abiraterone targets not only steroidogenesis but also directly antagonises AR activity.^30^ As 80% of ER+ breast cancer patients also express AR,^12,13^ we explored the notion that blocking AR signalling with abiraterone may provide benefit in ER+ breast cancer. Here, we provide mechanistic insights for the mode of action of abiraterone in ER+ breast cancer cell lines, modelling AI-sensitivity and resistant disease.

Our study revealed that abiraterone within the clinically achievable concentration range,^43,44^ had weak oestrogenic activity but contrasting effects on proliferation in endocrine sensitive vs. resistant breast cancer cell lines. More specifically, models of endocrine sensitive ER+ breast cancer showed increased proliferation and associated expression of G1/S checkpoint proteins indicative of cell cycle progression in response to abiraterone, whereas the reverse was true in the majority of cell lines modelling resistance to AI-therapy. This observation was independent of AR activity.

*ESR1* mutations are evident in ~20% of metastatic patients^45^ and appear enriched in response to AI therapy. Indeed, a recent study showed 39.1% of patients treated with AI therapy harboured an *ESR1* mutation in circulating tumour DNA and 49% of these were polyclonal.^46,47^ In order to explore the relevance of *ESR1* mutations in response to abiraterone, we used a panel of LTED cell lines that harboured naturally occurring ESR1^wt^, ESR1^Y537C^ or ESR1^Y537S^. Interestingly, SUM44-LTED, which express the *ESR1*^*Y537S*^ were the least responsive to the anti-proliferative effects of abiraterone. In contrast, LTED cell lines expressing *ESR1*^*wt*^ showed a marked reduction in proliferation with IC_50_ values over 30% lower than those seen for the *ESR1*^*Y537C*^ mutant cell line. Concordantly, cell death in response to abiraterone was more pronounced in both MCF7-LTED^wt^ and HCC1428-LTED compared with MCF7-LTED^Y537C^. Previously, we and others have shown that LTED models retaining ligand-independent ER activity are sensitive to high concentrations of E2, which conversely support proliferation of wt-MCF7 cells^31,38–41, 48^ a phenomenon which has been exploited clinically.^49^ The present study suggests that abiraterone may cause cell death in a similar manner to E2, although this warrants further investigation.

To explore the oestrogenic effect of abiraterone further, we assessed its impact on ER-mediated transactivation and ER-protein turnover. Abiraterone induced expression of oestrogen-regulated genes TFF1, GREB1 and PGR, which was also reflected in the enhanced recruitment of ER and CBP to target promoters. Of note, abiraterone caused proteasomal degradation of ER similar to that seen with E2, suggesting its ability to act as a ligand promoting ER activity. Taken together, this suggests that an increase in the protein turnover of ER might re-initiate growth in oestrogen-deprived MCF7 cells, whilst impacting negatively on the growth of MCF7-LTED cells, potentially inducing apoptosis.

Clinical studies exploring the inhibition of androgen production by abiraterone in patients who have relapsed on prior non-steroidal AI therapy have shown no added benefit compared with exemestane.^18^ In particular, there was no difference in the primary end point of PFS (50% PFS) between the abiraterone (3.7 months), exemestane (3.7 months) or the combination (4.5 months). One explanation for the failure of the trial was the apparent increase in progesterone, which may have provided a mitogenic stimulus bypassing the requirement for CYP17 blockade.^17,18^ Our data from the wt-MCF7, wt-HCC1428 and wt-SUM44 suggests that the combination of E2-deprivation and abiraterone treatment may enhance the agonist activity of abiraterone such that despite CYP17 blockade the drug itself can act as a ligand. Finally, the majority of patients within the trial received prior AI therapy and it is therefore likely that a large proportion harbour ESR1 mutations. Importantly, we showed LTED cells expressing ESR1^Y537S^ a common hot-spot mutation were resistant to abiraterone, whilst LTED cells containing ESR1^Y537C^ were responsive. Previous studies have shown the Y537S mutation enables helix 12 to undergo a conformational change exposing the AF2 cleft thereby facilitating recruitment of co-regulators in the absence of E2.^25,50^ Furthermore, ligand-binding assays showed lower affinity for E2, tamoxifen and ICI compared with ESR1^wt^.^50^ Hence we postulate abiraterone may not impact either directly or indirectly on the ability of ESR1^Y537S^ to drive proliferation. In the case of Y537C, it is unclear. It could hypothesised that as cysteine is a more reactive amino acid,^51^ it may impact on association of ESR1 with other proteins/cofactors and that binding of abiraterone may disrupt these interactions leading to cell death. Taken together, these data suggest the context in which resistance to AI-therapy is acquired may influence response to abiraterone highlighting once again the importance of correct patient selection.

## Electronic supplementary material

Table S1

Figure S1

Figure S2

Figure S3

Figure S4

supplementary Table and Figures
