# Supplementary material for: Abiraterone shows alternate activity in models of endocrine resistant and sensitive disease
Source: Br J Cancer. 2018 Jul 11;119(3):313–22. doi: 10.1038/s41416-018-0158-y (PMC6068155; doi:10.1038/s41416-018-0158-y)
Supplement: Supplementary file 1 — Table S1 [file 41416_2018_158_MOESM1_ESM.docx]

|  | **E2** | | **Abiraterone** | |
| --- | --- | --- | --- | --- |
|  | **Ki (nM)** | **95% CI** | **Ki (uM)** | **95% CI** |
| **wt-MCF7** | 0.16 | 0-1.58 | 0.39 | 0.1-1.2 |
| **MCF7-LTED^Y537C^** | 0.27 | 0.09-0.6 | 0.25 | 0-3.6 |
|  | **IC_50_ (nM)** | **95% CI** | **IC_50_ (uM)** | **95% CI** |
| **wt-MCF7** | 1.4 | 0.8-14.8 | 3.7 | 1-10.9 |
| **MCF7-LTED^Y537C^** | 2.5 | 0.8-5.9 | 2.4 | 0-33 |
