# Supplementary figures and images for: Abiraterone shows alternate activity in models of endocrine resistant and sensitive disease
Source: Br J Cancer. 2018 Jul 11;119(3):313–22. doi: 10.1038/s41416-018-0158-y (PMC6068155; doi:10.1038/s41416-018-0158-y)

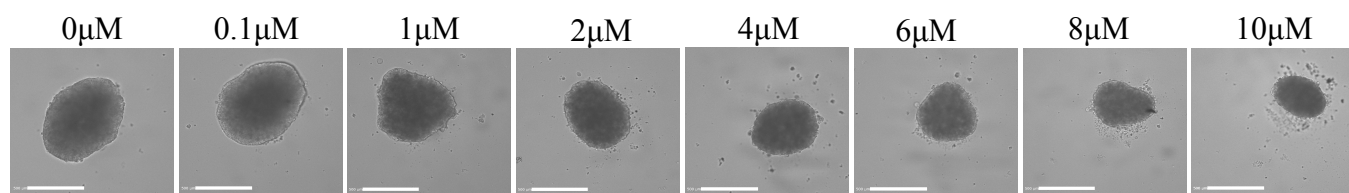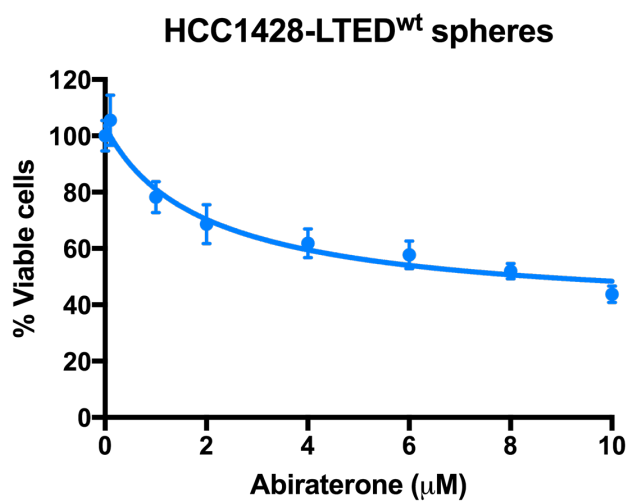

Supplement: Supplementary file 2 — Figure S1 [file 41416_2018_158_MOESM2_ESM.pdf]

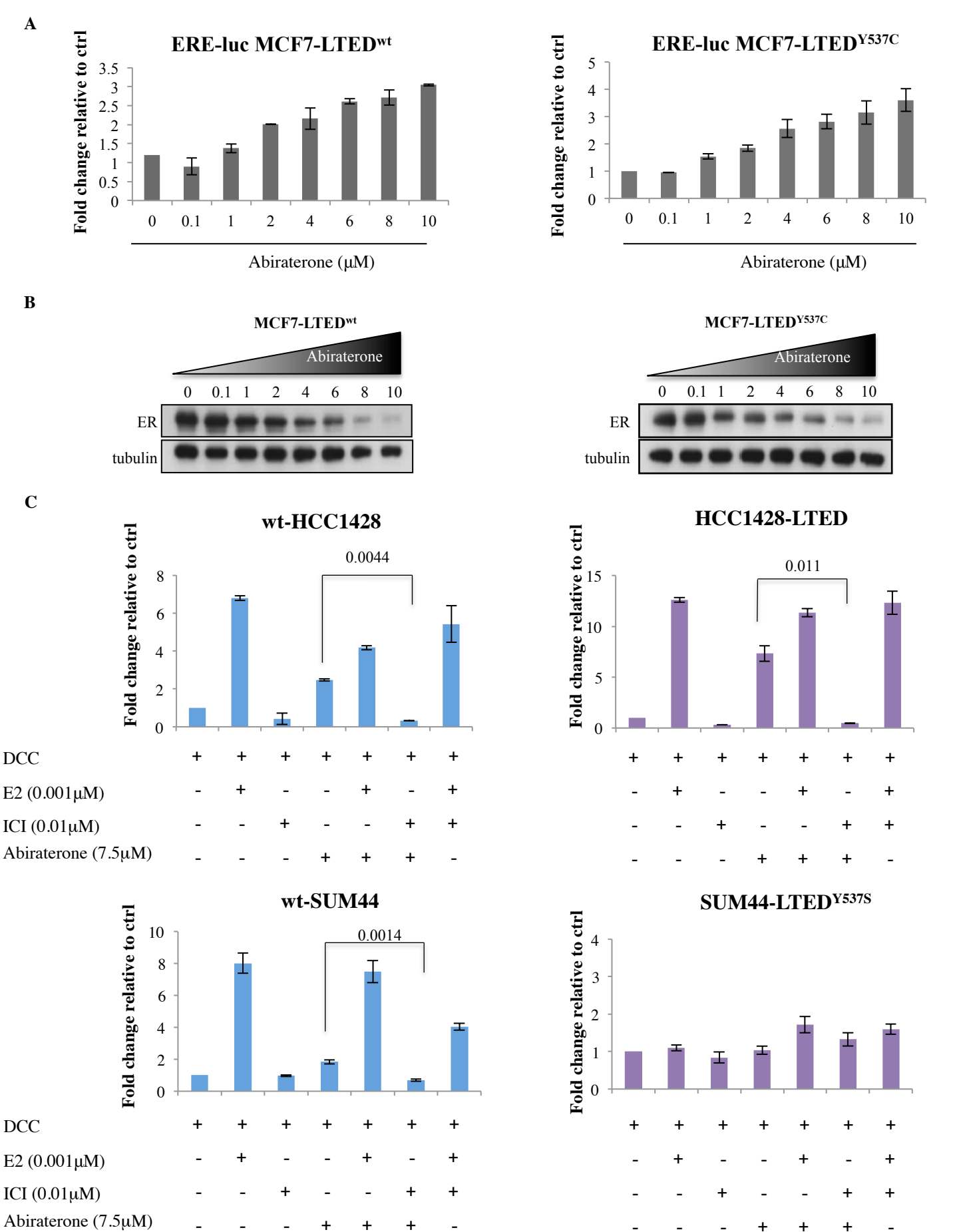

Supplementary Figure S2

Supplement: Supplementary file 3 — Figure S2 [file 41416_2018_158_MOESM3_ESM.pdf]

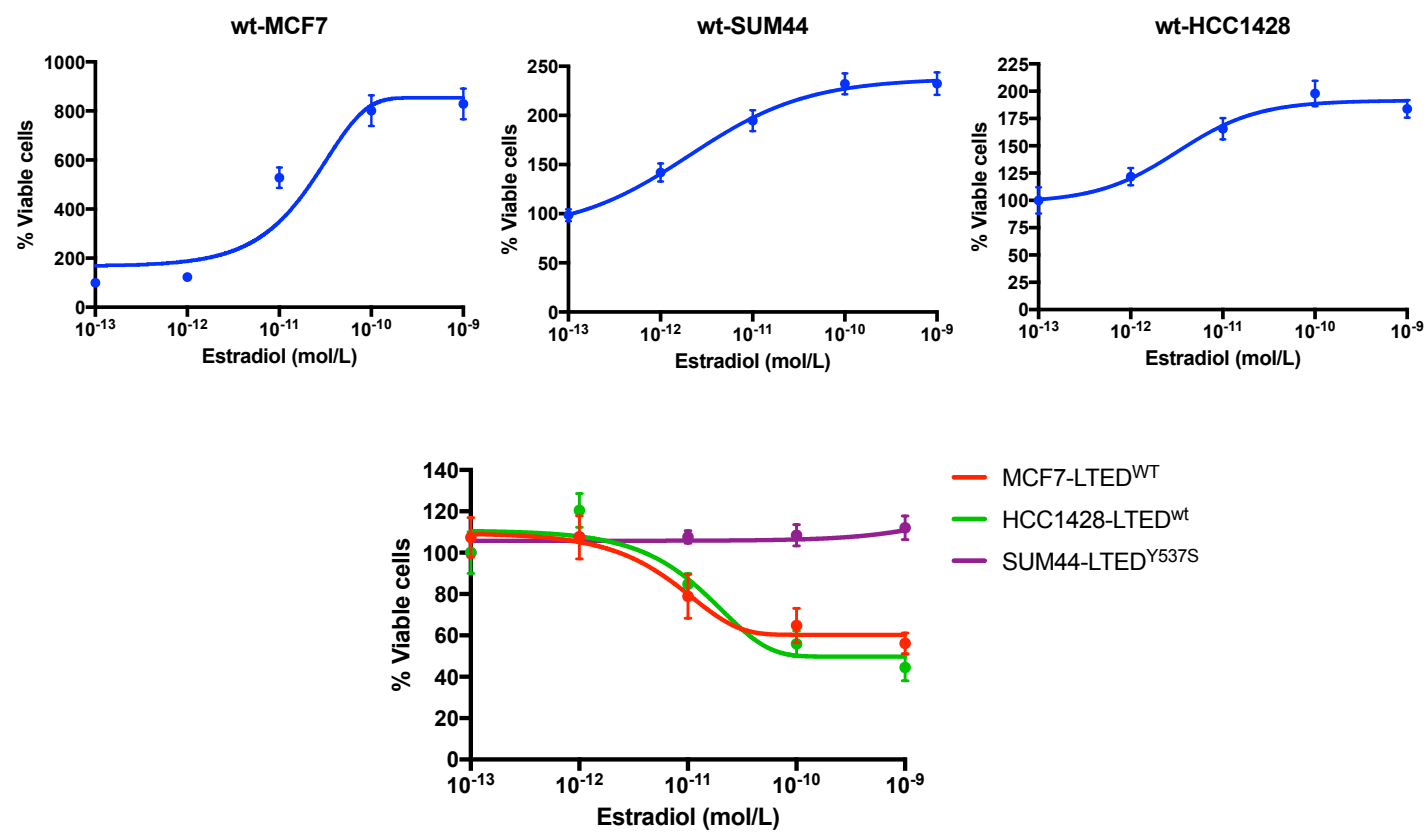

Supplement: Supplementary file 5 — Figure S4 [file 41416_2018_158_MOESM5_ESM.pdf]
