# Supplementary material for: Abiraterone shows alternate activity in models of endocrine resistant and sensitive disease
Source: Br J Cancer. 2018 Jul 11;119(3):313–22. doi: 10.1038/s41416-018-0158-y (PMC6068155; doi:10.1038/s41416-018-0158-y)
Supplement: Supplementary file 4 — Figure S3 [file 41416_2018_158_MOESM4_ESM.pptx]

## Slide 1
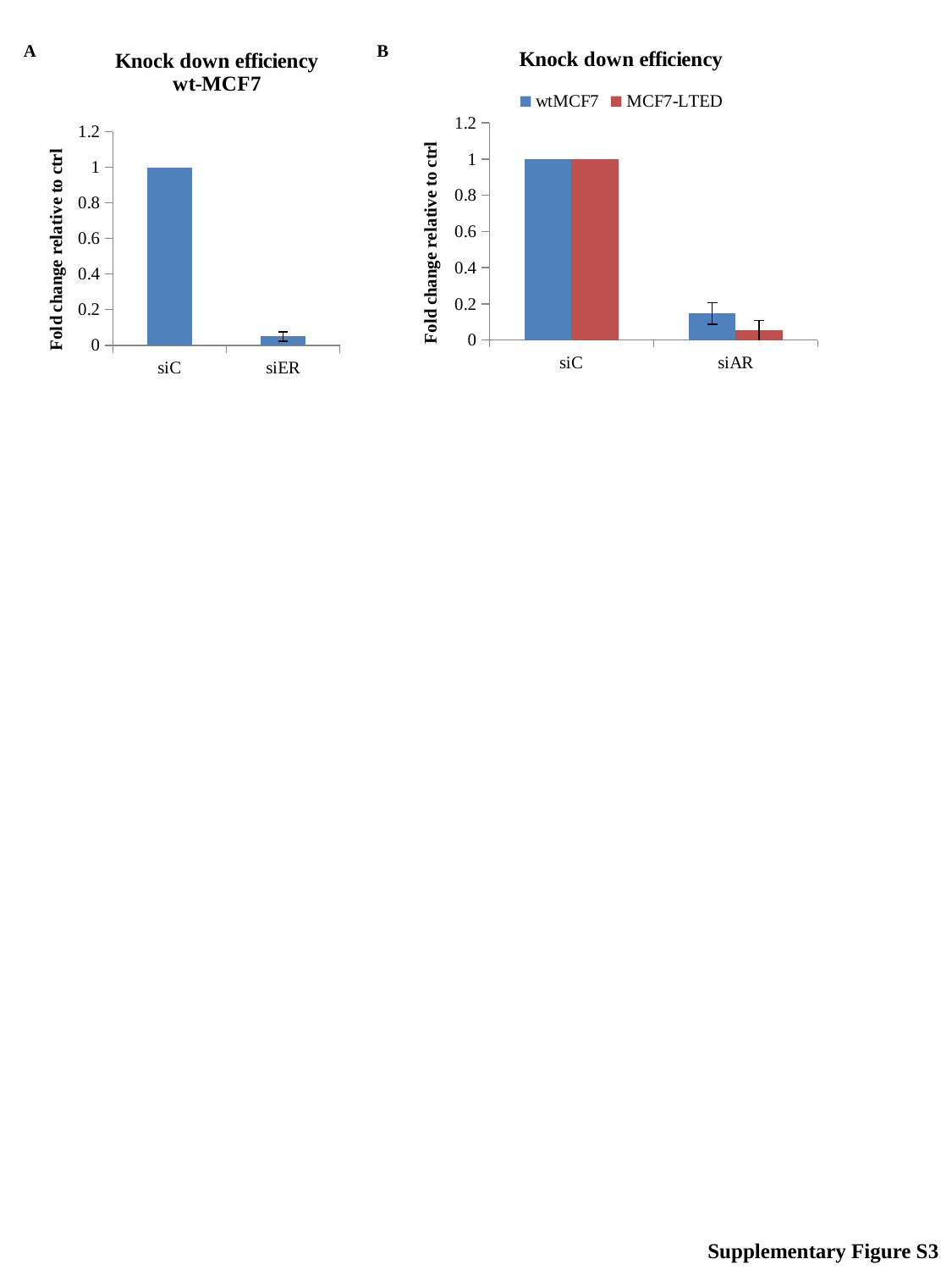

### Chart: Knock down efficiency
| Category | wtMCF7 | MCF7-LTED |
|---|---|---|
| siC | 1.0 | 1.0 |
| siAR | 0.146179886809177 | 0.0529154687281704 |A
B
### Chart: Knock down efficiency
wt-MCF7
| Category | |
|---|---|
| siC | 1.0 |
| siER | 0.0487883064247787 |Supplementary Figure S3
