# Supplementary material for: Abiraterone shows alternate activity in models of endocrine resistant and sensitive disease
Source: Br J Cancer. 2018 Jul 11;119(3):313–22. doi: 10.1038/s41416-018-0158-y (PMC6068155; doi:10.1038/s41416-018-0158-y)
Supplement: Supplementary file 6 — supplementary Table and Figures [file 41416_2018_158_MOESM6_ESM.docx]

**Supplementary Table S1. Ligand binding assay**

**Figure S1.** **Effect of abiraterone on proliferation assessed in 3D spheroids.** Images of HCC1428-LTED spheres in increasing concentrations of abiraterone accompanied with a viability assay of spheres. Bar equal 500 micrometres

**Figure S2.** **ER-mediated transactivation is increased in the presence of abiraterone in various AI-sensitive and AI-resistant ER+** breast cancer **cell line models.** **(A)** MCF7-LTED^wt^ and MCF7-LTED^Y537C^ were transfected with an ERE-luciferase (EREII-tk-luc) reporter construct and treated with increasing doses of abiraterone. Data shown is representative of two biological experiments and three replicates per treatment. Bars represent ± SEM. **(B)** Immunoblot assessment ER protein abundance after 48 hours treatment with escalating concentrations of abiraterone. **(C)** Cell lines (wt-HCC1428, HCC1428-LTED, wt-SUM44 and SUM44-LTED) were transfected with an ERE-luciferase (EREII-tk-luc) reporter construct and treated with E2, abiraterone, ICI 182,780 (ICI) alone or in combination. Data is representative of two biological experiments with three replicates for each treatment. Bars represent ± SEM.

**Figure S3. Efficiency of the knock down in the wt-MCF7 and MCF7-LTED^Y537C^. (A) si**ER and **(B) si**AR.

**Figure S4 Impact of escalating concentrations of E2 on Cell viability.** Wt-MCF7, wt-SUM44, wt-HCC1428, MCF7-LTED^wt^, SUM44-LTED^Y537S^, HCC1428-LTED were treated with increasing concentrations of E2 over 6 days. Cell viability was measured using TitreGlo and expressed as percent of viable cells relative to vehicle control.
